# Factors associated with asthma control: MOSAR study (Multicenter Observational Study of Asthma in Rabat-Morocco)

**DOI:** 10.1186/s12890-018-0624-6

**Published:** 2018-04-24

**Authors:** Imane Ghanname, Ahmed Chaker, Abha Cherkani Hassani, Laila Herrak, Serge Arnaul Ebongue, Mustapha Laine, Khalid Rahhali, Abdelhak Zoglat, Aida Maria Benitez Rexach, Samir Ahid, Yahia Cherrah

**Affiliations:** 1 Research team of Pharmacoepidemiology & Pharmacoeconomics, Laboratory of Pharmacology and Toxicology, Faculty of Medicine and Pharmacy, Mohammed V University, Rabat, Morocco; 2 Faculty of Health Sciences, International University of Casablanca, Bouskoura, Morocco; 3 Laboratory of Applied Mathematics, Faculty of Sciences, Mohammed V University, Rabat, Morocco; 4 Unit of training and research in Nutrition and Food Sciences, Faculty of Medicine and Pharmacy, Mohammed V University, Rabat, Morocco; 5 Department of Pneumology, Ibn Sina Hospital, Rabat, Morocco; 6 Department of Pneumology, Mohamed V Military Hospital, Rabat, Morocco; 7 Department of Pneumology, Moulay Youssef Hospital, Rabat, Morocco; 8 Department of Languages, International University of Casablanca, Bouskoura, Morocco; 9 Doctoral Student in Psychology, Walden University, Minneapolis, USA

**Keywords:** Asthma control, Epidemiology, Ordinal logistic regression, Proportional odds model, Protective factors, Risk factors

## Abstract

**Background:**

The purpose of the study is to describe the profile of patients with asthma and to identify the signifiant risks and the protective factors associated with asthma control.

**Methods:**

*A* prospective epidemiological study was conducted in three hospitals of Rabat-Morocco and included 396 patients with asthma. Differences in characteristics across the levels of asthma control were compared by the one-way analysis of variance for continuous variables, and chi-square test was used for categorical variables. The risk and protective factors associated with the asthma control levels were determined by Proportional Odds Model (POM) for bivariate and multivariate ordinal logistic regression, also expressed as Odds Ratios (OR) and 95% Confidence Intervals (95% CI).

**Results:**

From 7440 patients screened by 28 physicians, 396 were included in study. 53% of the particiants sufferd controlled, 18% had partly controlled and 29% had uncontrolled asthma symptoms. A multivariate ordinal logistic regression analysis showed that having respiratory infections (AOR = 5.71), suffering from concomitant diseases (AOR = 3.36) and being allergic to animals (AOR = 2.76) were positively associated with poor control of asthma. However, adherence to treatement (AOR = 0.07), possession of health insurance (AOR = 0.41) and having more than 2 children (AOR = 0.47) were associated with good asthma control.

**Conclusion:**

The study established a clinical-epidemiological profile of asthmatic patients in Rabat region in Morocco. By ordinal logistic regression we found that 6 factors - respiratory infections, concomitant diseases, animals allergy, adherence to treatment, health insurance and having more than two children – were associated with asthma control.

## Background

The World Health Organization estimates that there are now 235 million asthmatics worldwide [1] and more than 17 million of this individuals are in the United States [2]. In Morocco, 3.89% of the Moroccan population was affected by asthma, in 2009, which represents more than 1.2 millions people [3].

Prevalence of asthma varies, from 1 to 18% depending on the countries [4–9], its much cost, also its unpredictable evolution and the possibility to prevent its exacerbation, make asthma a capricious disease that forms a public health priority [10].

Despite the rapid progress of its knowledge, understanding the evolution of asthma is still insufficient. However, some deadly forms could be avoided through better supervision and better education of patients [2].

Studies in French populations showed there are 2000 deaths annually due to asthma, most of them could be avoided [2] and less asthma patient care often leads to severe forms that need hospitalization. Furthermore, nearly 50% of the asthmatic population does not take the prescribed treatment [11].

Current clinical practice displayed phenotypic characterization of asthma is difficult [12, 13]. Therefore, pragmatic decisions are necessary according to each patient [14]. A better understanding of the health status of each asthma patient and the evolution of this condition may provide (i) the appropriate patient prevention of the disease in adhering to prescriptions and medical advice, (ii) and aid clinicians to provide improved coping therapies and protocols to prevent adverse conditions leading to disease deterioration.

The study about the evaluation of anti-asthmatic drugs consumption in Morroco [15] demonstrated that despite its high prevalence [3], the therapeutic care of asthma remains unsatisfactory at the national level compared to other countries, probably due to a combination of factors; the very particular socioeconomic environment of the developing countries with its corollaries (low purchasing power ...); the part of the generic drug and the insubstantial health insurance which is poorly distributed; in addition to the inherent difficulties to asthmatic patients (influence beliefs) and the absence of national recommendations about asthma care.

This research aims to describe the clinical-epidemiological profile of asthmatic patients included in MOSAR study (**M**ulticenter **O**bservational **S**tudy of **A**sthma in **R**abat). Furthermore, check their asthma control levels and identify the major risks and the protective factors associated with asthma control in the actual clinical practice of the care of asthma in Morocco.

## Methods

### Study design

MOSAR (**M**ulticenter **O**bservational **S**tudy of **A**sthma in **R**abat) is a prospective observational study conducted on 42 months (from September 2010 to April 2014) in three hospitals of Rabat (Ibn Sina Hospital, Mohamed V Military Hospital and Moulay Youssef Hospital) on a sample of 396 asthmatic patients.

During this period, for each patient included in the study, physicians had to fill out a ad-hoc questionnaire, based on GINA guidelines and validated by experts, concerning asthma control level, sociodemographic, environmental and clinical characteristics and medications use. The date of inclusion in study is the date of the first contact with the patient.

A feasibility study was conducted in the three hospitals within the research to assess the validity and quality of the ad-hoc questionnaire.

### Study population

Among the consultants of pulmonology and allergology and according to GINA guidelines [16], adult patients were included in the study if they had a physician diagnosis of asthma for at least 3 months (to avoid entanglements with bronchiolitis [17]). The diagnosis was based on medical history, physical examination, and spirometry.

We have noticed across the different departments of pneumology in our study that the indivuduals who smoke more than 10 pack-years of cigarettes consulted were diagnosed with Chronic Obstructive Pulmonary Disease (COPD). We excluded them from our list automatically.

Patients with Bronchiectasis or Cardiac Asthma (a medical diagnosis of wheezing, coughing or shortness of breath due to congestive heart failure were also excluded from this study.

### Guidelines

GINA International guidelines [16] concern diagnosis, severity, treatment, monitoring and education of asthmatic patients.They permit adequate care management of the severity of asthma, with the aim of mastering the disease. The levels of severity and control were determined by combining several criteria: the frequency of diurnal and nocturnal symptoms, their impact on the activity and the sleep, and the results of lung function, use of inhaled short acting beta2-agonists (SABAs) and sever exacerbation [18].

### Ethics

The study protocol was approved by Ethics Committee for Biomedical Research (CERB) of the Faculty of Medicine and Pharmacy of Rabat performed in accordance with the Declaration of Helsinki, good clinical practice, and all relevant international and national legislations and written informed consent for participating in the study was also obtained from all participants.

### Variables

**The dependent variable** was asthma control. Patients were classified into 3 groups: controlled, partly controlled and uncontrolled asthma symptoms.

**The independent variables** were:

- *Socio-demographic variables:* age, sex, Body Mass Index (BMI), marital status, number of children, residence, educational level, occupation and health insurance.
- *Environmental and clinical variables:* season of consultation, reasons of visit, duration of asthma, family history of asthma, active and passive smoking, presence or absence of concomitant diseases, co-morbidities (rhinitis, allergic conjunctivitis, gastroesophageal reflux (GERD), respiratory infections), nocturnal awakenings, number of crisis, symptoms during 3 previous months (expectoration, wheezing, dyspnea, nocturnal coughing), presence or absence of allergies, lung function, and adherence to treatment.

### Statistical analysis

Continuous variables were described by mean and standard deviation (±*SD*) and categorical variables by absolute and relative frequencies. Differences in characteristics across the level of asthma control were compared by the one-way analysis of variance for continuous variables and a chi-square test was used for categorical variables. Values of *P* < 0.05 were considered significant in a bilateral approach.

The factors associated with the asthma control levels were determined by Proportional Odds Model (POM) for ordinal logistic regression. This regression model for ordinal data leading to the estimate of a single odds ratio that gives the risk to go from controlled to partly controlled asthma and from partly controlled to uncontrolled asthma (briefly “from controlled to uncontrolled asthma”).

A univariate ordinal logistic regression analysis was completed to separately examine the association of each factor with the asthma control levels (using controlled asthma as the reference category). Then, all factors with a statistical significance of *p*-value< 0.05 in the univariate analysis were included into a multivariate ordinal logistic regression.

The final models were built using stepwise selection factors method with a *p* < 0.05 significance level. The proportional odds assumption was tested with the Brant test (line parallelism), and the Hosmer-Lemeshow goodness-of-fit test was used to validate the model.

A database was automatically developed from the questionnaire in Form Designer of Epi Info software (version 3.5.1) and all statistical analysis were performed using SPSS (Statistical Package for the Social Sciences) (IBM 13.0 for Windows®).

## Results

**Fig. 1:**
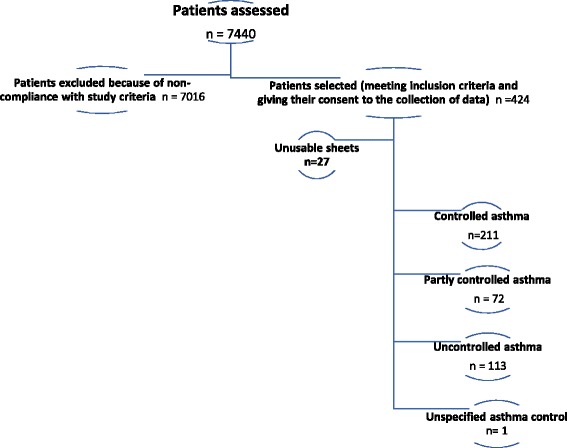
MOSAR study flow-chart

From September 2010 to April 2014, a total of 7440 patients visited the pneumology allergology services and were screened by 28 physicians. Only 396 patients (5.3% of the patients in pulmonology consultants for distinct reasons) met the inclusion criteria and were eligible for the study. Furthermore, among them, there were 211(53%), 72(18%) and 113(29%) patients who had respectively controlled, partly controlled and uncontrolled asthma symptoms (Fig. 1).

### Profile of patients with asthma

#### Socio-demographiccharacteristics

The socio-demographic characteristics of the study population were divided into three groups to represent the respective three levels of asthma control, presented in tables.

The socio-demographic factors that differed significantly with asthma control levels were: age, civil status, number of children and health insurance (Table 1).

**Table 1:** Socio-demographic characteristics of the studypopulationaccording to levels of asthma control

	All	Controlled	Partly controlled	Uncontrolled	*p-value*
	*n* = 396	*n* = 211(53%)	*n* = 72(18%)	*n* = 113(29%)	
Age, n(%)					.005*
[18–39] years	162(42.0)	69(42.6)	39(24.1)	54(33.3)	
[40–64] years	198(51.3)	120(60.6)	27(13.6)	51(25.8)	
> 64 years	26(6.7)	17(65.4)	2(7.7)	7(26.9)	
Female gender, n(%)	266(67.3)	139(52.3)	44(16.5)	83(31.2)	.191
BMI, km/m^2^, n(%)					.304
Normal weight or underweight (BMI < 25)	99(45.8)	60(60.6)	15(15.2)	24(24.2)	
Overweight (BMI [25–30])	69(31.9)	34(49.3)	15(21.7)	20(29.0)	
Obesity (BMI > 30)	48(22.2)	33(68.8)	6(12.5)	9(18.8)	
Married, n(%)					.025*
No	90(23.3)	37(41.1)	22(24.4)	31(34.4)	
Yes	296(76.7)	168(56.8)	46(15.5)	82(27.7)	
Number of children, n(%)					.002*
≤2 children	147(45.2)	67(45.6)	30(20.4)	50(34.0)	
> 2 children	178(54.8)	116(65.2)	25(14.0)	37(20.8)	
Place of residence, n(%)					.544
Large town	317(85.4)	175(55.2)	52(16.4)	90(28.4)	
Small town	44(11.9)	22(50.0)	9(20.5)	13(29.5)	
Village	10(2.7)	3(30.0)	3(20.0)	4(40.0)	
Educational level, n(%)					.113
Secondary education or University	94(30.3)	42(44.7)	18(19.1)	34(36.2)	
No scooling or primary school	216(69.7)	123(56.9)	37(17.1)	56(25.9)	
Habitual activity, n(%)					.712
Activelyemployed	94(26.4)	48(51.1)	18(19.1)	28(29.8)	
Retired	23(6.5)	15(65.2)	4(17.4)	4(17.4)	
Housework	221(62.1)	126(57.0)	32(14.5)	63(28.5)	
Student	18(5.1)	8(44.4)	4(22.2)	6(33.3)	
Social Health insurance, n(%)					.000*
No	92(23.6)	28(30.4)	24(26.1)	40(43.5)	
Yes	298(76.4)	180(60.4)	47(15.8)	71(23.8)	

*BMI* Body Mass Index, *n* number of cases, *SD* Standard Deviation, *%* percentage

Results are presented as mean ± SD or n(%) when appropriate

*Significant, *P* < 0.05

The average age was 42 years and more than half of the patients (51.3%) were between 40 and 64 years old; higher proportion of older patients were found in the controlled asthma group.

76.7% of patients were married and the ratio of married patients varied significantly across the three control levels from 15.5% in partly controlled to 27.7% and 56.8% in uncontrolled and controlled asthma respectively (*p* = 0.025). Among the health insured patient population, 60.4% of the participants were considered as controlled, 15.8% and 23.8% as partly controlled and uncontrolled respectively (*p* < 0.05).

No differences were showed on sex, BMI, the place of residence, educational level, and occupation among the three groups of patients (Table 1). Also, 266 of patients were females (67.3%) and the majority were in the categorized under the uncontrolled asthma group, conversely to men (31.2% versus 23.3%). Furthermore, less than the half had an average weight or were underweight (45.8%) and about 22.2% were obese. Moreover, 69.7% were illiterate or had primary school level and 30.3% had a secondary or higher educational level, and more significant part of the patient population were houseworker (62.1%).

#### Environmental and clinical factors

The clinical and ecological factors that differed significantly with asthma control levels were: reasons of the current visit, smoking, concomitant diseases, co-morbidities (rhinitis, conjunctivitis, GERD, and respiratory infections), number of crisis, and symptoms during 3 previous months (expectoration, wheezing, dyspnea) and allergy to animals (Table 2).

**Table 2:** Environmental and clinical characteristics of the study population according to levels of asthma control

	All	Controlled	Partly controlled	Uncontrolled	*p-value*
	*n* = 396	*n* = 211	*n* = 72	*n* = 113	
Season of consultation, n(%)					.280
Summer	69(17.5)	44(63.8)	10(14.5)	15(21.7)	
Automn	129(32.7)	62(48.1)	26(20.2)	41(31.8)	
Winter	155(39.3)	82(52.9)	25(16.1)	48(31.0)	
Spring	41(10.4)	21(51.2)	11(26.8)	9(22.0)	
Reasons of the current visit, n(%)					.000*
A follow-up visit	348(87.9)	208(59.8)	60(17.2)	80(23.0)	
Aggravation of asthma	48(12.1)	3(6.2)	12(25.0)	33(68.8)	
History of asthma, mean(±SD)	11.4(**±**9.7)	12(**±**9.9)	10(**±**9.5)	11(**±**9.5)	.331
Family history of asthma, n(%)					.514
None	226(58.2)	115(50.9)	42(18.6)	69(30.5)	
Yes (parents, grandparents, uncles and aunts)	162(41.8)	92(56.8)	26(16.0)	44(27.2)	
Smoking habits, n(%)					.031*
Never smoker	327(83.4)	166(50.8)	57(17.4)	104(31.8)	
Active smoker	35(8.9)	21(60.0)	9(25.7)	5(14.3)	
Ex-smoker	30(7.7)	22(73.3)	4(13.3)	4(13.3)	
Passive smoking, n(%)					.881
No	144(39.5)	77(53.3)	27(18.8)	40(27.8)	
Yes	221(60.5)	120(54.3)	37(16.7)	64(29.0)	
Concomitant diseases, n(%)					.001*
No	136(34.4)	89(65.4)	21(15.4)	26(19.1)	
Yes	259(65.6)	121(46.7)	51(19.7)	87(33.6)	
Co-morbidities, n(%)					
Rhinitis	221(59.7)	109(49.3)	44(19.9)	68(30.8)	.045*
Allergic conjunctivitis	165(44.6)	78(47.3)	34(20.6)	53(32.1)	.039*
Gastroesophageal Reflux Disease	38(9.6)	12(31.6)	8(21.1)	18(47.4)	.011*
Respiratory Infections	65(16.5)	13(20.0)	18(27.7)	34(52.3)	.000*
Waking during the night, n(%)					.000*
No	254(65.3)	205(80.7)	18(7.1)	31(12.2)	
Yes	135(34.7)	0(0.0)	53(39.3)	82(60.7)	
Number of crisis, n(%)					.000*
≤2/week	276(70.6)	208(75.4)	55(19.9)	13(4.7)	
> 2/week	115(29.4)	0(0.0)	16(13.9)	99(86.1)	
Symptoms during 3 previous months					
Expectoration	29(7.3)	0(0.0)	7(24.1)	22(75.9)	.000*
Wheezing	92(72.4)	0(0.0)	36(39.1)	56(60.9)	.016*
Dyspnea	109(27.5)	0(0.0)	38(34.9)	71(65.1)	.000*
Nocturnal coughing	69(55.2)	0(0.0)	26(37.7)	43(62.3)	.283
Allergy to, n(%)					
Dust	295(76.8)	153(51.9)	56(19.0)	86(29.2)	.199
Animals	76(19.8)	29(38.2)	18(23.7)	29(38.2)	.008*
Mold	233(60.7)	123(52.8)	41(17.6)	69(29.6)	.767
Pollen	121(31.6)	57(47.1)	24(19.8)	40(33.1)	.157
Smoke	20(5.1)	9(45.0)	6(30.0)	5(25.0)	.375
Medication	40(10.1)	25(62.5)	3(7.5)	12(30.0)	.168
Certain foods	49(14.2)	25(51.0)	6(12.2)	18(36.7)	.333
Season variation	89(22.5)	50(56.2)	11(12.4)	28(31.5)	.260
Other cause of crisis, n(%)					
Stressful event	8(2.0)	4(50.0)	2(25.0)	2(25.0)	.879
Emotion	2(0.5)	0(0.0)	0(0.0)	2(100.0)	.081
Effort	16(4.1)	6(37.5)	3(18.8)	7(43.8)	.348
Pulmonary function testing (FEV), n(%)					.377
FEV < 80%	29(64.4)	16(55.2)	7(24.1)	6(20.7)	
FEV ≥80%	16(35.6)	6(37.5)	7(43.8)	3(18.8)	
Adherence to treatment, n(%)					.488
No	12(3.0)	6(50.0)	1(8.3)	5(41.7)	
Yes	384(97.0)	205(53.4)	71(18.5)	108(28.1)	

*FEV* Forced Expiratory Volume in one second, *n* number of cases, *SD* Standard Deviation;%: percentage

Results are presented as mean ± SD or n(%) when appropriate

* Significant, *P* < 0.05

In fact, 48 (12.1%) of patients had emergency visits because of an exacerbation of their asthma. The rate of distribution of aggravation through the groups of patients increased in a statistically significant way from 6.2% in controlled to 25% and 68.8% in partly and uncontrolled asthma respectively.

Around 259 (65.6%) of patients had concomitant diseases, and its proportion differed significantly across the groups of patients with the higher value in controlled asthma group (46.7%). As well, Rhinitis (59.7%) and allergic conjunctivitis (44.6%) were the most representative co-morbidities. The proportion of each co-morbiditie was statistically significantl for the groups of patients with the higher value in uncontrolled asthma group for GERD (47.4%) and respiratory infections (52.3%) and in controlled asthma group for rhinitis (49.3%) and allergic conjunctivitis (47.3%).

Indeed, 35% of patients would wake up during the night (60.7% were uncontrolled and 39.3% partly controlled) and 29.4% had more than two crisis in a week with 86.1% in uncontrolled asthma. The proportion of expectoration, wheezing and dyspnea symptom during the 3 previous months, with the exception of noctucnal coughing, was higher and statistically significant in uncontrolled asthma. Farthermore, 19.8% of patients suffered from an allergy to animals with lower values in the partly controlled asthma group (23.7%).

Moreover, 83.4% of the patients in the study had never smoked, whereas 7.7% were ex-smokers. Also, only 35 of 392 patients were active smokers (represented by occasional smokers, and Light smoker) and most of them (60%) with a controlled asthma.

As outlined in Table 2, the three groups of patients did differ concerning season of consultation, history of asthma, family history of asthma, passive smoking, nocturnal coughing and pulmonary function test. The mean duration of asthma was 18 years (±12.9) and 66% of patients were diagnosed more than 10 years ago. Furthermore, Pulmonary function tests were performed in only 11% of patients at the inclusion visit.

Interrogation alone is not enough to appreciate patient compliance, and we are deprived in this assessment unless using methods unbeknownst to the patient that ethics has forbidden. All patients received a therapeutic education. Poor adherence was reported in 8.3% of the included population. Failure to obey to the prescription dosage and treatment duration, especially in Ramadan, irregular consultations, unsuitable inhalation system, without neglecting the socioeconomic context namely the purchasing power; that does not often allows patients to acquire the treatment; adherence to medical coverage and the high burden of other concomitant chronic diseases were the leading causes.

### Factors associated with asthma control

#### Univariate analysis

Univariate ordinal logistic regression analysis using the asthma control as the dependent variable and all socio-demographic, environmental and clinical factors as independent variables are reported in Table 3.

**Table 3:** Factors in the univariate ordered logistic regression analysis for the respective asthma control categories

	Unadjusted OR	95% CI	*p-value*
1. Socio-demographic factors			
Age (Referent^a^: [18–39])			.008
[40–64] years	0.55	[0.37–0.83]	.004*
> 64 years	0.48	[0.20–1.11]	.085
Sex (Referent^a^: male)	1.23	[0.82–5.00]	.312
BMI (Referent^a^: normal weight or underweight)			.131
Overweight	1.47	[0.81–2.63]	.204
Obesity	0.70	[0.34–1.43]	.327
Married	0.61	[0.39–0.95]	.029*
Number of children (Referent^a^: ≤2)	0.47	[0.30–0.71]	.000*
Place of residence (Referent^a^: large town)			.384
Small town	1.17	[0.64–2.13]	.618
Village	2.14	[0.66–7.14]	.207
Educational level (Referent^a^:secondaryor university)	0.61	[0.39–0.97]	.037*
Occupation (Referent^a^: actively employed)			.483
Retired	0.55	[0.22–1.39]	.209
Housework	0.84	[0.53–1.33]	.453
Student	1.24	[0.48–3.23]	.656
Health insurance	0.34	[0.22–0.53]	.000*
2. Environmental and clinical factors			
Season of consultation(Referent^a^: summer)			.218
Automn	1.83	[1.03–3.23]	.040*
Winter	1.60	[0.91–2, 78]	.103
Spring	1.44	[0.67–3.03]	.349
History of asthma	0.99	[0.97–1.01]	.267
Family history of asthma	0.81	[0.55–1.19]	.283
Smoking habits(Referent^a^: never smoker)			.015*
Active smoker	0.59	[0.29–1.19]	.140
Ex-smoker	0.36	[0.16–0.83]	.017*
Passive smoking	1.00	[0.67–1.5]	.993
Concomitant diseases	2.15	[1.43–3.23]	.000*
Co-morbidities			
Rhinitis	1.66	[1.11–2.5]	.014*
Allergic conjunctivitis	1.65	[1.11–2.44]	.013*
Gastroesophageal reflux	2.56	[1.37–4.76]	.003*
Respiratory Infections	4.21	[2.50–7.14]	.000*
Allergy to:			
Dust	1.40	[0.88–2.22]	.161
Animals	2.01	[1.25–3.23]	.004*
Mold	1.16	[0.78–1.72]	.469
Pollen	1.47	[0.97–2.22]	.066
Season variation	0.96	[0.61–1.52]	.862
Adherence to treatment	0.71	[0.24–2.08]	.042*

*CI* Confidence Interval, *OR* Odds Ratio. ^*^Significant, P < 0.05; ^a^Reference category (OR = 1) is no answer

Among the sociodemographic factors, the categories “being 40-64 years ols” (OR: 0.55; 95% CI [0.37; 0.83]), “having more than two children” (OR: 0.47; 95% CI [0.30; 0.71]), “having none or primary educational level” (OR:0.61; 95% CI [0.39; 0.97]) and “having a health insurance” (OR: 0.34; 95% CI [0.22; 0.53]) have a significant negative association with the dependent category,in fact they are protective factors for “Uncontrolled asthma”, in other words, these factors decrease the risk to have an “uncontrolled asthma” (OR < 1 and *p*-value < 0.05). Also, being married (OR: 0.61; 95% CI [0.36; 0.95]) was associated with lower risk factors and better health status, even in the presence of many confounding effects. However, the remaining factors, sex, BMI, place of residence and occupation were not associated with asthma control in univariate analysis.

Among the environmental and clinical factors, having concomitant diseases (OR: 2.15; 95% CI [1.43; 3.23]), having rhinitis (OR: 1.66; 95% CI [1.11; 2.5]), having conjunctivitis (OR: 1.65; 95% CI [1.11; 2.44]), having gastroesopheal reflux (OR: 2.56; 95% CI [1.37; 4.76]) and having respiratory infections (OR: 4.21; 95% CI [2.50; 7.14]) were found as a risk factors of asthma (OR > 1 and *p*-value < 0.05). The protective factors for uncontrolled asthma was adherence to treatment (OR: 0.71; 95% CI [0.24; 2.08]). In contrast, the season for the consultation, the patient and family history of asthma, passive smoking and allergies to dust, mold, pollen or season variation were not associated with asthma control in univariate analysis.

#### Multivariate analysis

The final models were built using multivariable ordinal logistic regression with stepwise selection factors method with asthma control as a dependent variable and as well as independent variables associated with asthma control in the univariate analysis. Table 4 represents the results of the multivariate analysis. Adjusted odds ratio and corresponding 95% confidence intervals were calculated.

**Table 4:** Factors in the multivariate ordered logistic regression analysis for the respective asthma control categories

	Adjusted OR	95% CI	*p-value*
1. Socio-demographic factors			
Age (Referent^a^: [18–39])			
[40–64] years	0.75	[0.38–1.49]	.414
> 64 years	0.77	[0.21–2.76]	.683
Married	2.37	[0.63–8.85]	.201
Number of children (Referent^a^: ≤2)	0.47	[0.24–0.93]	.030*
Educational level (Referent^a^: secondary or university)	0.50	[0.24–1.04]	.064
Health insurance	0.41	[0.18–0.93]	.033*
2. Environmental and clinical factors			
Smoking (Referent^a^: never smoker)			
Active smoker	0.51	[0.19–1.39]	.191
Ex smoker	0.50	[0.16–1.60]	.242
Concomitant diseases	3.36	[0.99–11.34]	.050*
Co-morbidities			
Rhinitis	0.84	[0.29–2.45]	.756
Allergic conjunctivitis	1.00	[0.49–2.03]	1.00
Gastroesophageal Reflux Disease	1.83	[0.70–4.82]	.219
Respiratory Infections	5.71	[2.39–13.63]	.000*
Allergy to animals	2.76	[1.36–5.59]	.005*
Adherence to treatment	0.07	[0.01–0.98]	.049*

*CI* Confidence Interval, *OR* Odds Ratio, *Significant, *P* < 0.05; ^a^Reference category (OR = 1) is no answer

In this model, patients having respiratory infections (Adjusted-OR: 5.71; 95% CI [2.39; 13.63]), having concomitant diseases (Adjusted-OR: 3.36; 95% CI [0.99; 11.34]), allergic to animals (Adjusted-OR: 2.76, 95% CI [1.36; 5.59], those adhered to treatment (Adjusted-OR: 0.07; 95% CI [0.01; 0.98]), having health insurance (Adjusted-OR: 0.41; 95% CI [0.18; 0.93]) and having more than two children (Adjusted-OR: 0.47; 95% CI [0.24; 0.93]) remained significantly associated with ordered asthma control levels (all with *p*-value< 0.05).

However, no statistical association with asthma control was found regarding age, civil status, educational level, smoking, having rhinitis, GERD or conjunctivitis (all with *p*-value > 0.05).

## Discussion

MOSAR study allows us to identify the asthmatic profile of the patients referred to the pneumology consultation of three hospitals in Rabat. Our study showed an asthmatic percentage among consultants for other reasons of 5.7% higher than the prevalence of asthma in Morocco (3.89%) found by Benkheder et al. in the Maghreb study AIRMAG [19], which may be explained by the centralization of specialized consultation in the big cities,but resembling other countries with a permanent and continuous rise of this prevalence [20–22].

The study showed by Stempel et al. [23] which was focused on the fluctuation control of asthmatic patients over a three years period estimated that the majority of patients presented uncontroled signs during the duration of the disease. Cazzoletti et al. [24] reported in their European study on asthma control that the rate of uncontrolled patients ranged from 20% (Iceland) to 67% (Italy).Almost two-third of asthmatic patients included in a study evaluating the degree of asthma control in Spain in 2006 were poorly controlled [25]. More recently, Vervloet et al. found a high rate of asthmatic patients with inadequate control of asthma in real life despite the fact that 95% of patients were treated with anti-asthmatics [26]. Our study revealsed that 29% of patients had uncontrolled asthma.

The female dominance observed in our sample of patients was also reported in the European cohort study ENFUMOSA [27] and by many authors in the Maghreb and elsewhere [3, 28–31].

Asthma affects all ages with varying prevalences from country to another. The average age of our patients was 42 years, with no significant difference between various control levels, was similar to the levels found in most studies [19, 30, 32]. In our sample of patients, the age group most affected was between 40 and 64 years (198patients (51.3%)); in the French decennial survey, INSEE [28] it is rather beyond this age where there is a higher prevalence.

More than half of our patients were overweight or obese (54.1%), but seems to note have influence on the asthma control level.The relationship between overweight and asthma was identified by analysis combining data from cohort studies; the risk of asthma was increased for men and for overweightwomen as well.Asthma risk is multiplied by 1.4 in overweight people without obesity and 1.9 in obese people [33].

Coogan et al. [22] found that a higher BMI is often associated with asthma and correlates with poor control, On the other hand, a cohort of 1265 patients in Sutherland et coll [34]. showed that obesity related to a more inadequate response to treatment than non-obese patients.

Nevertheless, there is still controversy to determine if asthma is secondary to obesity or if asthma and obesity are the result of conventional genetic factors, diet or physical inactivity [33, 34].

Loerbroks et al. [35] reported in a cohort of 5114 patients of 40 to 65 years old that asthma is associated with obesity and its prevalence is higher for obese women.

In our study, the prevalence of active smokers (8.9%) was lower than that found in the literature where it ranges from 15 to 25% depending on the country [36, 37]. However, more than half of our patients undergoing passive smoking (60.5%). This could be explained by two facts: first, the Moroccan socio-cultural context, where there is under-reporting of smoking for women and a relatively low prevalence (3.1% versus 31.5% for men according to Nejarri et al.) [38]; second, that smokers who are prone to develop asthma either stop smoking because of respiratory symptoms before the diagnosis is made, or continue to smoke at a moderate rate because of their respiratory symptoms [39].

The active or passive smoking, was described as a risk factor for the onset of asthma and reduced control [1, 22, 40–45]. Moreover, it can alter the response to inhaled corticosteroids [46].

Three-quarters of our patients (72%) had been consulted in autumn or winter; the high humidity could explain this in the city of Rabat. Similarly, according to a Belgian investigation [47], 5.1% of households in the Brussels region say they were embarrassed at home in the last 12 months of the study by a moisture problem.

This combination of moisture and the prevalence of asthma symptoms was explained. The proliferation of mites or molds is one of the possible explanations for this relationship [48].

While some countries such as France do not know any difference between the prevalence of asthma with the seasons [28]; there is a resurgence of crises during the pollen season for others [28, 49, 50].

According to Apter et al. [42] atopy is often related to asthma. This relationship is due to development of societies [51].

In our study the presence of at least one concomitant disease was found in more than half of patients (65.6%).Allergic rhinitis was predominantly represented (59.7%) and was three times higher than the French decenal investigation (21.6%) [10].

In the ATHMOS study by Vervloet and al. [26] confirm that symptoms of rhinitis were associated with uncontrolled asthma that was previously shown by Barros et al [52] Additionally, the presence of rhinitis predicted the development of asthma [53]. In our study, rhinitis was associated with controlled asthma. However, patients with allergic rhinits in MOSAR study were treated according to ARIA guidelines, and untreated patients or non-compliance patients still risk factor for uncontrolled asthma. This was also described by Wallaert which reports that well treated rhinitis improves asthma control [54].

In more than half of our patients with at least one comorbidity, we found a good asthma control, while Apter and others reported the opposite in their articles [42, 55, 56].

The GERD was found in 9.6% of patients. Although it is usually considered as risk factor and poor asthma control [57], for Didier et al. [58], it is not justified to be present if there are no digestive symptoms.

Only the one-third of our patients reported nocturnal crisis, while the majority of patients had less than two attacks per week, this can be interpreted by the high number of patients having controlled asthma in our sample.

The measuring of the FEV (Forced Expiratory Volume in one second) was not possible in 89% of the patients, due to poor clinical conditions or difficulties to measure; 35.6% have a normal FEV.

Asthma duration remains underestimated (recall bias), and it often represents a risk of error [30] relatively short for most of our patients (11.4 ± 9.7 years) in comparison with the study of Allegra et al. [17] conducted in Italy in 2011 (16.9 ± 13.4) years.

Pulmonary radiography is requested in the initial exploration to define diagnosis, but it is not a usual follow-up examination for asthma patients. By against, spirometry, is an examination which occupies an essential place both in the diagnosis and in the monitoring asthma [16], and should regulary be performed or at the request to assess the impact of asthma on lung function.

None of our patients had done laboratory tests, such as the rate of eosinophils in the blood or sputum; these tests were not routine examinations [59].

Therefore, the diagnosis of asthma was based on the presence of clinical symptoms as given in the latest Global Initiative of Asthma guidelines (GINA guidelines) [16]. Dyspnea, Nocturnal coughing and wheezing were the symptoms found in our patients, in accordance with the review of literature [28].

The goal of asthma management is to achieve and maintain its control; knowing that the control level is fluctuating [23], our study only reflects its trend during the 42 months of our research.

According to the World Health Organization [3], asthma is incurable. However, adequate support allows to curb the disorder and gives the asthmatic patient a better quality of life, a appropriate care, especially for the comorbidities, rhinitis, GERD and respiratory infections associated with asthma,improves control level.

Asthma management remains unsteadily at the national plan compared to other countries [15, 60]. The reasons for the inadequate quality of treatment is due to the combination of two factors; firstly the socioeconomic environment very particular developing countries with its outcomes: low purchasing power, inadequate health insurance and on the other hand the difficulties inherent in asthma patients: influence beliefs, poor adherence ..., and other extenuating factors.

We note that some factors circumstances associated with the level of control have not been discussed or evaluated in this work during our investigation. Exploration by the flowmeter (rudimentary gesture amounting respiratory function), the psychological impact and the doctor-patient relationship, therapeutic patient education (which develop asthma as recurrent acute illness and not as a chronic disease [61], household income that may be the leading cause of non-adherence were not taken in consideration for the study.

**Fig. 2:**
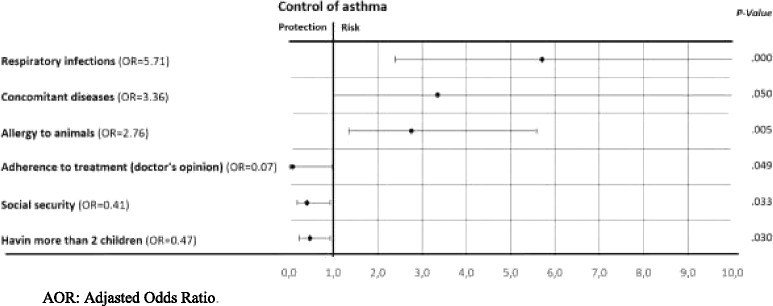
Risk and protective factors for uncontrolled asthma

Multivariate ordinal regression analysis identified several independent factors associated with asthma control as shown in Fig. 2. Having respiratory infections, having concomitant diseases and to be allergic to animals were found as risk factors for asthma.

Patients with respiratory infections had 5.7 times more risk of having worse asthma control (uncontrolled or partly controlled asthma), Those with concomitant diseases showed 3.4 times more risk and those allergic to animals had 2.8 times more.

In contrast, adhered to treatment, having health insurance and having more than two children were found as protective factors for uncontrolled asthma. Adherence to treatment significantly increased the odds of being in controlled asthma category by 7%. Regarding health insurance, patients having health insurance showed a better control of asthma, their odds to have controlled asthma was 40% times higher than those without controlled asthma. Finally, patients having more than 2 children were 50% more likely to have a controlled asthma compared with those having 2 or fewer children.

As limitation of the study, the absence of lung functions and chest RX could have impact on the trust of diagnosis. Radiography, it is not considered as essential test for asthma diagnosis according to GINA.

Also, FEV1 is not required for all patients but it alteration is considered for future risk. Howeve when we talk about control we mean clinical control.

## Conclusion

The study has established a clinical-epidemiological profile of asthmatic patients in Rabat region-Morocco and examined the socio-demographic, environmental and clinical factors associated with asthma control in the region. Proportional odds model for ordinal logistic regression was used to identify possible contributory factors. The results of the study show that factors such as respiratory infections, concomitant diseases and animals allergy were perceived as risk factors for asthma. In contrast, adherence to treatment, health insurance and having more than two children was observed as protective factors for uncontrolled asthma.

This work constitutes a blank for the profiling and the asthma phenotype in Rabat region awaiting a generalization of the results by work nationally.

## Abbreviations

AOR: Adjusted Odds Ratio
BMI: Body Mass Index
FEV: Forced Expiratory Volume in one second
GER: GastroEsopheal Reflux
GERD: Gastroesophageal Reflux Disease
GINA: Global INitiative of Asthma
OR: Odds Ratio
POM: Proportional Odds Model
SD: Standard Deviation
